# Formulation of a Synergistic Enzyme Cocktail for Controlled Degradation of Sorghum Grain Pericarp

**DOI:** 10.3390/foods12020306

**Published:** 2023-01-09

**Authors:** N. U. Sruthi, Pavuluri Srinivasa Rao, Sarita Jane Bennett, Rewati Raman Bhattarai

**Affiliations:** 1 School of Molecular and Life Sciences, Faculty of Science and Engineering, Curtin University, Bentley 6102, Australia; 2 Agricultural and Food Engineering Department, Indian Institute of Technology Kharagpur, Kharagpur 721302, India

**Keywords:** sorghum, enzyme synergism, pericarp, morphology, crystallinity

## Abstract

Sorghum is one of the major grains produced worldwide for food and fodder, owing to its nutritional profile advantages. However, the utilisation of whole grain sorghum as an ingredient in conventional food formulations is limited due to its poor digestibility, which requires the removal of the outer fibrous layers. Grain breakage and loss of essential nutrients also disadvantage traditional milling practices. Using carbohydrate degrading enzymes to hydrolyse the grain pericarp is a novel approach to biopolishing, where selective degradation of the pericarp layers occurs without adversely affecting the nutrient profile. A collective synergism of enzymes has been proven to cause effective hydrolysis compared to individual enzymes due to the complex presence of non-starch polysaccharides in the grain’s outer layers, which comprise a variety of sugars that show specific degradation with respect to each enzyme. The present study aimed to formulate such an enzyme cocktail with xylanase, cellulase, and pectinase in different proportions for hydrolysing sorghum grain pericarp by determining the yield of specific sugars in the pericarp extract after a certain period of incubation. The results showed that the xylanase enzyme has a major effect on the grain bran composition compared to cellulase and pectinase; however, a synergistic mixture yielded more hydrolysed sugars and anti-nutrients in the extract compared to each of the enzymes individually. The results were confirmed by morphological and crystallinity studies of the soaked grain. Compared to conventional water-soaked samples, grains soaked in a cocktail with 66.7% xylanase, 16.7% cellulase, and 16.7% pectinase had visibly thinner and more degraded fibre layers.

## 1. Introduction

Sorghum (*Sorghum bicolor* L.) is a staple food crop in the semi-arid and tropical regions of the world. It is predominantly grown on the Asian and African continents due to its climatic resilience and adaptability to various adverse environmental conditions [1]. It is one of the most prominent crops produced worldwide, ranking 5th after maize, wheat, rice, and barley, with an annual production of 58.7 million tons [2]. On top of its remarkable agronomic performance, sorghum contains a unique phenolic profile distinct from major cereals, with abundant and diverse phenolic components [3,4]. It is also a rich source of macro- and micro-nutrients, primarily starch, fat, protein, fat-soluble vitamins, fand such as D, E, and K, vitamin B, phytosterols, carotenoids, and non-starch polysaccharides (NSPs) [5]. Sorghum has also been shown to positively affect nutrition in people diagnosed with celiac disease, diabetes, and obesity [6]. Furthermore, the polyphenols present in sorghum have been found to possess a higher ability to suppress the proliferation of cancer cells than the array of phenols present in other food sources, suggesting that sorghum grain can serve as an efficient and economic edible supplement in cancer treatment [7,8].

On account of its climatic resilience and superior health benefits, there has been significant interest in sorghum and its value-added products from academia and food industries. There have been attempts to incorporate sorghum as a functional food constituent in a variety of products such as bread, pasta, noodles, cookies, snacks, tea, and weaning foods to improve their quality and nutritional benefits [9,10,11,12,13]. However, the sorghum pericarp is composed of NSPs and anti-nutrients such as phytates and tannins, the leading causes of insolubility and resistance in sorghum, making dehulling highly desirable [14]. Decortication is a primary processing step for sorghum as it removes most of the grain pericarp and testa layers, eliminating the antinutrients and increasing the bioavailability of proteins. Additionally, decortication can also improve the consumer acceptance of sorghum products, in particular palatability, as sorghum genotypes with tannins are reported to have a bitter and astringent off-flavour [15]. The most efficient method is achieved when maximum removal of the outer pericarp, testa, and germ occurs with maximum endosperm recovery and minimum breakage, which can be completed either manually or mechanically.

The traditionally practiced hand pounding method is a labour-intensive and tedious process that yields non-uniform grain output with varied efficiency. Mechanical decortication, employing abrasive rollers and emery stones, significantly affects the composition of the polished grain due to the increased exposure to friction on the contact surfaces of the equipment [16]. Additionally, a small seed size, friable pericarp, a prominent integral germ, and an extremely variable endosperm texture add mechanical polishing difficulty [17]. An alternative to traditionally practiced mechanical decortication and other pre-treatments is to use biocatalysts to degrade outer cellulosic layers. Microorganisms with GRAS (generally regarded as safe) status such as some fungi are adept at producing highly heterogenous enzymes specialised in plant cell wall degradation, including glycosyl-hydrolases, oxido reductases, lyases, and esterases. These are also categorised under the category of carbohydrate-active enzymes and are further divided into families in accordance with the amino acid sequence and structural interaction [18]. Enzymes including cellulases, xylanases, pectinases, and proteases, amongst others, interact with the grain pericarp layers and selectively degrade the pericarp by the hydrolysis of different glycosidic, covalent, and hydrolytic bonds, thus requiring less intense further decortication resulting in more palatable and healthier products [19]. Enzymes have been used both individually and in combination as a pre-treatment prior to mechanical decortication and milling for grains such as rice [20], wheat [21], pigeon pea [22], and corn [23] to minimise the loss as well as to improve the quality of the obtained yield. This reiterates the potential of an enzymatic treatment to loosen the pericarp of the sorghum. A synergistic combination of enzymes can improve the hydrolysis process. Synergism can only be ascertained when the enzyme cocktail-induced release of the target products surpasses the amount of the products released when the enzymes are applied individually in the same amount as in the cocktail [24]. The present study aims to formulate an enzyme cocktail with xylanase, cellulase, and pectinase with a synergistic impact on the degradation of fibrous pericarp layers of sorghum grain to aid in the process of mechanical decortication.

## 2. Materials and Methods

### 2.1. Raw Material and Chemicals

Whole sorghum grain (M35-1, Mahindra male) was acquired from the Indian Institute of Millet Research, Hyderabad, India. The grains were cleaned and sieved using a destoner cum grader cum aspirator (DGA S3500, Perfura Industries, Tamil Nadu, India) to eliminate impurities and were stored in steel drums at 75% RH and 30 °C until further processing. The original moisture content was 10 ± 0.22%.

The commercial enzymes used in the study were xylanase (ex. *Aspergillus niger*; 2.5 U/mg), cellulase (plant culture tested; 10 U/mg), and pectinase (ex. *Aspergillus niger*; 8 U/mg) obtained from Himedia and SRL with manufacturer provided activities. These were chosen based on the NSPs and water unextractable cell wall materials of the grain pericarp. The chemicals and reagents used were procured from SRL, Himedia, Spectrochem, and Merck, including methanol, 3,5-dinitrosalicylic acid (DNS) reagent, crystalline phenol, sodium sulphite, Rochelle salt, phloroglucinol, glacial acetic acid, carbazole reagent, vanillin, ammonium iron (III) sulphate, bipyridine, thioglycolic acid, hydrochloric acid, and sodium hydroxide.

### 2.2. Sample Preparation and Enzyme Treatment

The cleaned and sorted sorghum grains (10 g) were soaked in 20 mL acetate buffer (0.1 M, pH 5) with proportions of enzymes added as described in Section 2.3. The overall concentration of the enzyme cocktail was fixed at 50 mg/100 g dry matter based on preliminary studies (unpublished data) to ascertain the effect of individual enzymes and their combinations on grain composition. The time of incubation and temperature were kept constant at 45 °C and 6 h, which encompasses the recommended operating conditions of the three selected enzymes. The mixture of grain and enzyme solutions was refrigerated at 5 °C following the 6 h incubation period to arrest further enzyme activity. The grains were strained, rinsed five times with distilled water, and shade-dried in a single layer until the desired moisture content for decortication (12.5 ± 0.1%) was reached. The dried grains were kept in a desiccator containing silica gel to avoid any moisture gain prior to morphological and microstructural analysis. The grains were examined for morphological and structural changes, and the pericarp extract was analysed for various released components from the grain.

### 2.3. Experimental Mixture Design

**Figure 1:**
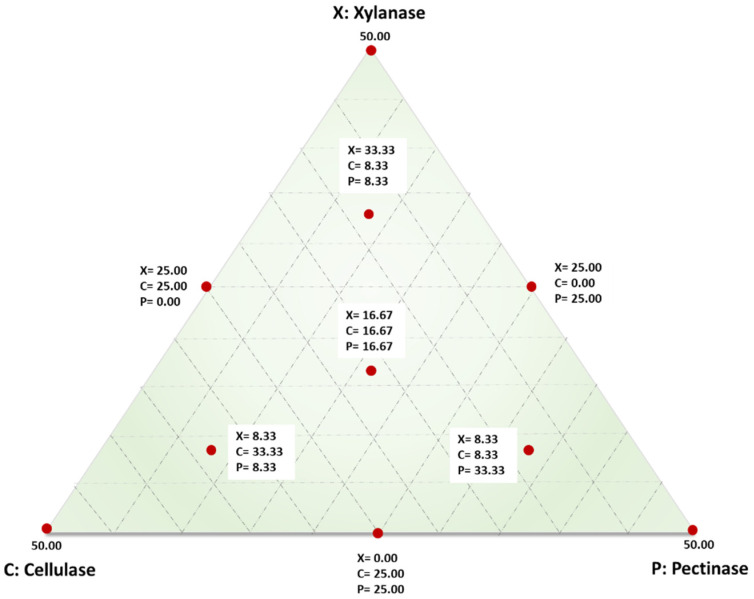
Points augmented simplex lattice mixture design for enzyme cocktail formulations comprising of xylanase (X), cellulase (C), and pectinase (P) with the mixture sum at the vertices representing the overall cocktail concentration of 50 mg/100 g dry matter.

An analysis of variance (ANOVA) based on a simplex-lattice mixture design with response surface methodology (RSM) was employed to analyse the data (Design Expert 10.0.1.0 (Stat ease Inc., Minneapolis, MN, USA)). The results were used to formulate the composition of individual enzymes in the cocktail to attain maximum degradation of sorghum grain pericarp for minimising the intensity of further required decortication. The individual concentrations (mg/100 g dry matter) of xylanase (X), cellulase (C), and pectinase (P) were selected as independent variables, and the concentration (mg/g) of pericarp components released into the buffer was the response, namely, total soluble solids (°Bx), total reducing sugars, glucose, xylose, galactouronic acid, total condensed tannin, and total phytate. The proportion of components was presented as fractions of the mixture with a sum (X + C + P) of 50. The experimental design showing the different combinations and concentrations of the three enzymes is presented in Table 1, with ten different experimental enzyme combinations (runs), each run repeated twice. The run order was randomised to lessen the influence of any unexplained variability. The ten runs were three single-enzyme treatments (vertex points), three two-enzyme mixtures (half-way points), and four three-enzyme mixtures (one centre point and three six-quarter points) (Figure 1). Best fit equations were developed for all the responses, and multiple response optimisations were performed using Design Expert 10.0.1.0 to identify the best proportion of enzymes in the cocktail. The grains soaked in water for 6 h were treated as the control to compare the effect of enzyme tempering to conventional water soaking.

### 2.4. Biochemical Analyses

The estimated data were expressed as mean ± standard deviation, and each measurement was done in triplicate.

#### 2.4.1. Proximate and Mineral Composition

The moisture content of grains was determined following the method AOAC-935.29 [25]. Whole grains were then decorticated in a field level decorticator (GP 3350, Perfura Industries, Tamil Nadu, India) to obtain polished grains. The grains, both whole and decorticated, were then ground to flour in a rotary mill (Pulverisette 14.702, Fritsch GmbH, Idar-Oberstein, Germany) and sieved to obtain flour with a particle size of not more than 425 μ for further compositional analysis. The gravimetric method was used to obtain the ash content following the method AOAC-923.03 [25]. The protein content was determined from the organic nitrogen concentration according to the Kjeldahl method following the method AOAC-979.09 [25]. The total lipid analysis was carried out in a soxhlet apparatus (SOCS PLUS, Pelican, India) by extracting 5 g flour with hexane according to the method AOAC-2003.06 [25]. The amount of crude fibre was determined using an automatic crude fibre analyser (FIBRA PLUS, Pelican, Chennai, India) following the method AOAC-962.09 [25]. A semi-automated system (Fibertec™ 1023, Foss, India) was used to estimate the dietary fibre content of in a 1 g flour sample according to the method AOAC-991.43 using the enzymatic gravimetric method. The carbohydrate content was estimated by the difference method [26]. Iron and zinc contents were estimated by digesting 0.5 g sample in a microwave digestion with conc. nitric acid at 170 °C for 30 min and then subjecting it to atomic absorption spectrometry (ICE3500, Thermo Fisher Scientific, Waltham, MA, USA) by running iron and zinc standards.

#### 2.4.2. Total Soluble Solids

Total soluble solids (TSS; °Brix) of the extracts obtained from partial hydrolysis of sorghum grain with the enzyme cocktail, hereafter referred to as the sample extract, were determined using a hand-held refractometer (PAL-1, Atago, Tokyo, Japan) after calibration with distilled water according to the AOAC method 932.12 [25].

#### 2.4.3. Reducing Sugars

The dinitrosalicylic (DNS) method was followed for estimating the content of reducing sugars (RS) in the sample extract as per Kapoor et al. with slight modifications [27]. In brief, to 0.5 mL of the sample extract, 3 mL DNS reagent and 2.5 mL distilled water were added and kept in a boiling water bath for 5 min. While the contents were still warm, 1 mL of 40% Rochelle salt solution was added. The solution was allowed to cool and the absorbance was read at 540 nm against the blank, where glucose was used as the standard to plot the calibration curve.

#### 2.4.4. Glucose

For in vitro quantitative determination of glucose, a commercially available Autospan GOPOD (glucose, oxidase, and peroxidase) test kit was used, which was purchased from ARKRAY Healthcare Pvt. Ltd., Mumbai, India. and the prescribed procedure was followed.

#### 2.4.5. Xylose

The determination of xylose (D-xylose) was completed using a phloroglucinol assay [28]. The method involves the formulation of a colour agent by dissolving 0.5 g phloroglucinol in a mixture of 100 mL glacial acetic acid and 10 mL concentrated hydrochloric acid (HCl). To 0.5 mL of the sample extract, 5 mL of the prepared reagent was added and the mixture was heated at 100 °C for 4 min. The contents were instantly cooled to room temperature under running tap water and the absorbance was noted at 554 nm against a blank containing water in place of the sample and D-xylose standard.

#### 2.4.6. Galacturonic Acid

The carbazole-sulphuric acid method was used to estimate galacturonic acid (GalA) content in the extract, following the method by Yang et al. [29] with slight modifications as described. To 200 µL of the sample extract, 3 mL concentrated sulfuric acid and 100 µL carbazole reagent were added. The contents were thoroughly mixed and incubated at 60 °C for 60 min, following which it was cooled. The absorbance was measured at 530 nm with water as a blank. GalA solutions (10–80 g/mL) were used as the standard.

#### 2.4.7. Total Condensed Tannins

The total condensed tannin (TCT) in the sample extract was determined by the vanillin-HCl assay described by Kumar and Rao [30]. To 0.25 mL of the sample extract, 1.5 mL 4% vanillin in methanol and 0.75 mL concentrated HCl were added. The contents were then incubated at room temperature for 20 min and the absorbance was measured at 500 nm with catechin as the standard.

#### 2.4.8. Total Phytate

The phytate content in the extract was determined as per Buddrick et al. [31]. Briefly, 0.5 mL of the extract and 2 mL ferric solution (0.2 g ammonium iron (III) sulphate mixed in 100 mL 2 N HCl and volume made up to 1000 mL with distilled water) were mixed and the contents were incubated in a boiling water bath for 30 min. After cooling the mixture using ice water, 2 mL of 2, 2′-bipyridyl solution (10 g 2, 2′-bipyridine in 10 mL thioglycollic acid made up to 1000 mL using distilled water) was added, and the absorbance was read immediately at 519 nm against a water blank with phytate phosphorus as the standard.

### 2.5. Statistical Analyses and Optimisation

The development of the mixture design, statistical analysis, modelling of experimental data, response surface generation, desirability functional analysis, and optimisation were completed using Design Expert 10.0.1.0 statistical software. Each set of observations was modelled to respond to the factors assessed at each experimental point as generated by the design. Models for analysing data in mixture designs were generally obtained by a combination of Scheffé-type models for the mixture factors and traditional response surface models for the design variables [32]. The following polynomial special cubic (Equation (1)) and quadratic (Equation (2)) models were fitted to the data:

(1) $$Y=\overset{q}{\underset{i=1}{\sum }}\beta_{i}x_{i}+\overset{q-1}{\underset{i<j}{\sum }}\overset{q}{\underset{j}{\sum }}\beta_{ij}x_{i}x_{j}+\overset{q-2}{\underset{i<j}{\sum }}\overset{q-1}{\underset{j<k}{\sum }}\overset{q}{\underset{k}{\sum }}\beta_{ijk}x_{i}x_{j}x_{k}\;$$

(2) $$Y=\overset{q}{\underset{i=1}{\sum }}\beta_{i}x_{i}+\overset{q-1}{\underset{i<j}{\sum }}\overset{q}{\underset{j}{\sum }}\beta_{ij}x_{i}x_{j}\;$$

where *Y* is the estimated response variable, *β_i_*, *β_ij_*, and *β_ijk_* are the constant coefficients of mixture variables produced by the prediction models for linear and non-linear terms, and *x_i_*, *x_j_*, and *x_k_* are the coded concentrations for each mixture component. The adjusted R^2^ and *p*-values were used to explain the statistical significance of the models. The variances were considered statistically significant at *p* < 0.05. Tukey’s honestly significant difference (HSD) was applied post hoc to evaluate the significant differences between the mean values using SPSS 20 (IBM SPSS Statistics, USA), setting the level of significance at *p* < 0.05. The optimisation was calculated by employing the desirability function combining all the optimisation targets into one (desirability) function [33]. The mathematical expression used to describe desirability is shown in Equation (3):

(3) $$D={\left(\Pi_{i=1}^{n}d_{i}\right)}^{\frac{1}{n}}={\left(d_{1}\times d_{2}\times d_{3}\ldots \ldots \ldots \times d_{n}\right)}^{\frac{1}{n}}\;$$

where *D* is the desirability function, d_i_ represents the desirability of each individual response ranging from 1 to n, and n is the number of responses being optimised.

### 2.6. Time Course of Enzymatic Hydrolysis

For determining the time course release of pericarp components, aliquots were taken at 1 h intervals for 6 h, immediately refrigerated at 5 °C, centrifuged, filtered, and analysed for TSS, RS, glucose, xylose, GalA, TCT, and total phytates. Two combinations which yielded the maximum and minimum final yield were chosen for comparison along with extract obtained upon water soaking.

### 2.7. Characterisation Techniques

#### 2.7.1. Scanning Electron Microscopy

The surface structure of enzyme-tempered, water-soaked, and untreated sorghum grain was analysed using scanning electron microscopy (EVO 60 with Oxford EDS Detector, Carl ZEISS SMT, Roßdorf, Germany) under high vacuum conditions and a maximum acceleration voltage of 30 kV. The analysed samples were cut in half transversally under sterile conditions. Halved grains were then placed onto aluminium stubs using double-sided carbon conducting tape with the fractures facing down, and were sputter-coated with gold using a vacuum sputter coater (CA76, POLARON-SC7620, POLARON, Watford, UK). The lateral side of the outer grain surface was observed through the microscope at a range of magnifications.

#### 2.7.2. X-ray Diffraction (XRD) Pattern

The enzyme-tempered, water-soaked, and untreated grains were packed into separate crucibles and pushed down using a stainless-steel weight. The crystalline properties and XRD patterns of the different samples were observed using an X-ray diffractometer (D2 PHASER, Bruker, Germany) equipped with copper kα filtered radiation, operating at 30 kV voltage and with a filament current of 10 mA. Signals of the reflection angle 2θ from 2° to 60° were recorded at a scanning speed of 2°/min, which covers all the considerable sample crystallites. The diffraction peaks were smoothed and analysed using the OriginPro 8.5 software package.

## 3. Results and Discussion

The experimental matrix of the quadratic simplex lattice mixture design is shown in Table 1, along with the results for the response variables (released pericarp constituents) for each assay. The enzyme activities of each individual enzyme for different treatment combinations are given in Table A1.

### 3.1. Proximate and Mineral Composition

The flour from whole and decorticated sorghum grains was analysed for proximate and mineral composition and the results obtained are shown in Table 2. The obtained composition of whole grain falls in the range of previous results found in the literature [26,34]. However, the differences obtained could be explained as a result of the varietal difference and cultivation conditions. Significant changes (*p* < 0.05) were observed in the composition of whole and decorticated grain flours and the values differ with respect to the initial moisture content of the grain and time of polishing, as previously reported [16]. It was seen that carbohydrates form the major grain constituent of sorghum in both whole and decorticated fractions, with the latter showing a greater amount as the starch content in the endosperm was concentrated upon removal of the peripheral parts of the grain.

However, the protein, fat, ash, crude fibre, and dietary fibre contents of the decorticated grain were reduced by 52.5%, 20.3%, 34.7%, 23.9%, and 27.7%, respectively, compared to the whole grain. This can be accredited to the removal of the hull, separation of germ, and a reduction in cellulosic layers upon decortication. A significant reduction was also observed in the decorticated grain’s iron and zinc contents, as the latter, in particular, is mostly located in the peripheral layers leading to greater removal [35]. The reduction in polyphenols and anti-nutrients, as presented in Table 2, can offer nutritional advantages pertaining to increased bioavailability of minerals and protein [36], and has previously been reported by Hama et al. [35] and Galán et al. [37], where significant losses were observed in the sorghum grain composition following decortication. The results also confirm that a major portion of macro- and micro-nutrients are concentrated in the grain outer layers, leading to a significant reduction during decortication that demands the need for novel grain pre-treatments or decortication techniques to minimise this loss and improve the decortication efficiency.

### 3.2. Biochemical Analysis of Pericarp Extract

**Figure A1:**
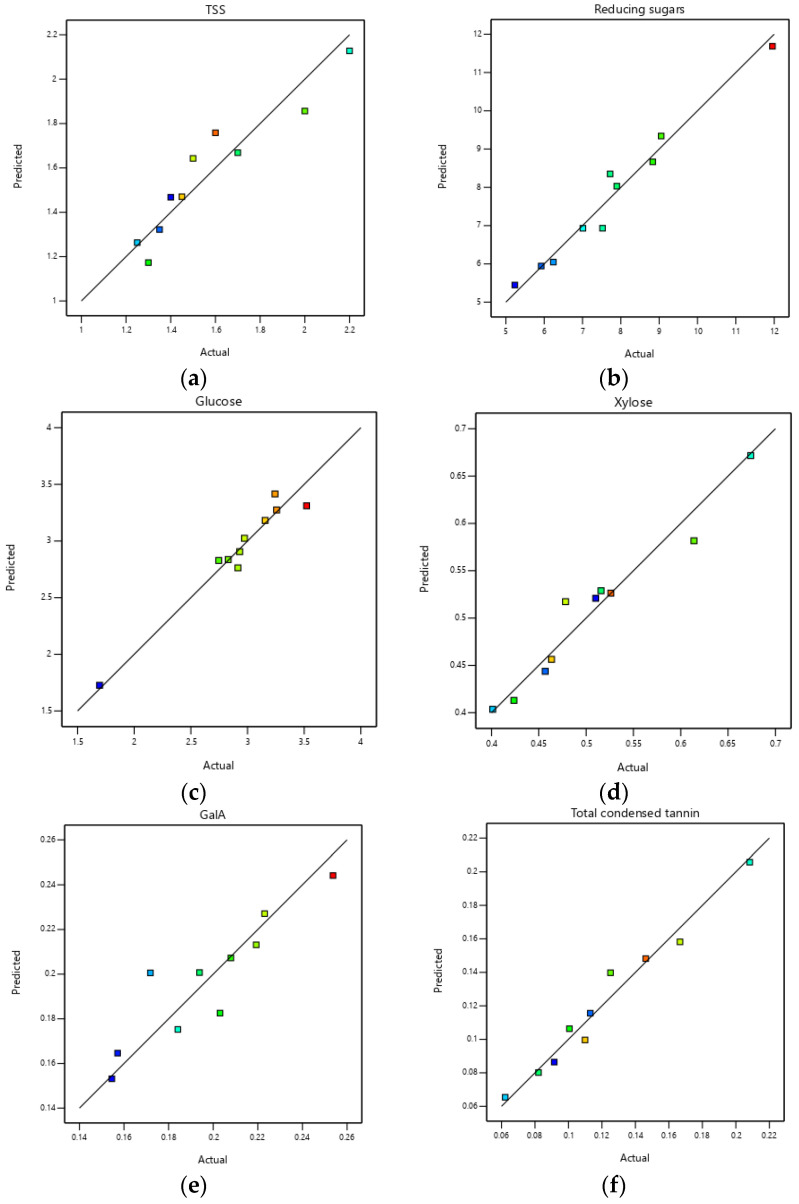

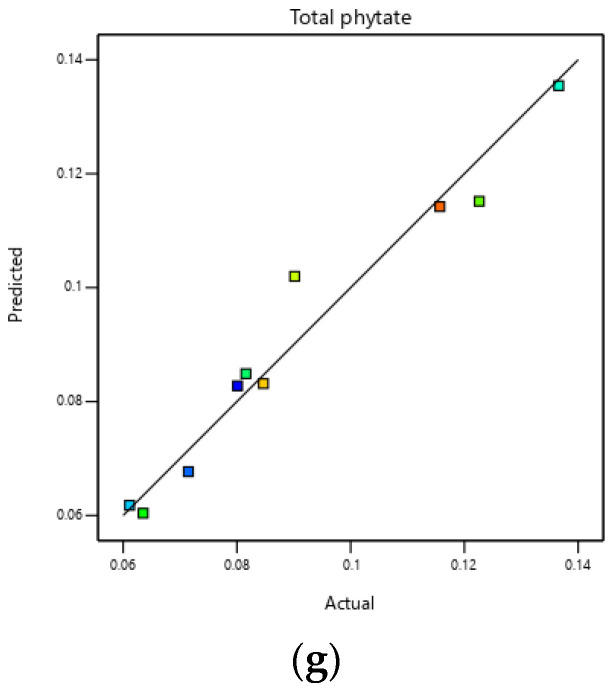
Scatter plots showing the relationship between the predicted and actual values of each response in the regression model: (**a**) TSS, (**b**) reducing sugars, (**c**) glucose, (**d**) xylose, (**e**) GalA, (**f**), total condensed tannins, and (**g**) total phytate.

Soaking sorghum grains with an enzyme cocktail of different cell wall degrading enzymes, as described in Section 2.3, was used to obtain extracts with hydrolysed pericarp components that are thought to be rich in sugars and phenols [38]. Table 1 presents the concentration of each component obtained in the extract under different treatment conditions. Table 3 presents the results of the regression analysis and ANOVA for all the models obtained for different responses. These coefficients indicate the importance of each component in the mixture in linear as well as interactive effects. Figure A1 (Appendix B) shows the scatter plots showing the quality of the developed model by comparing the predicted and the actual values in the regression model.

**Figure 2:**
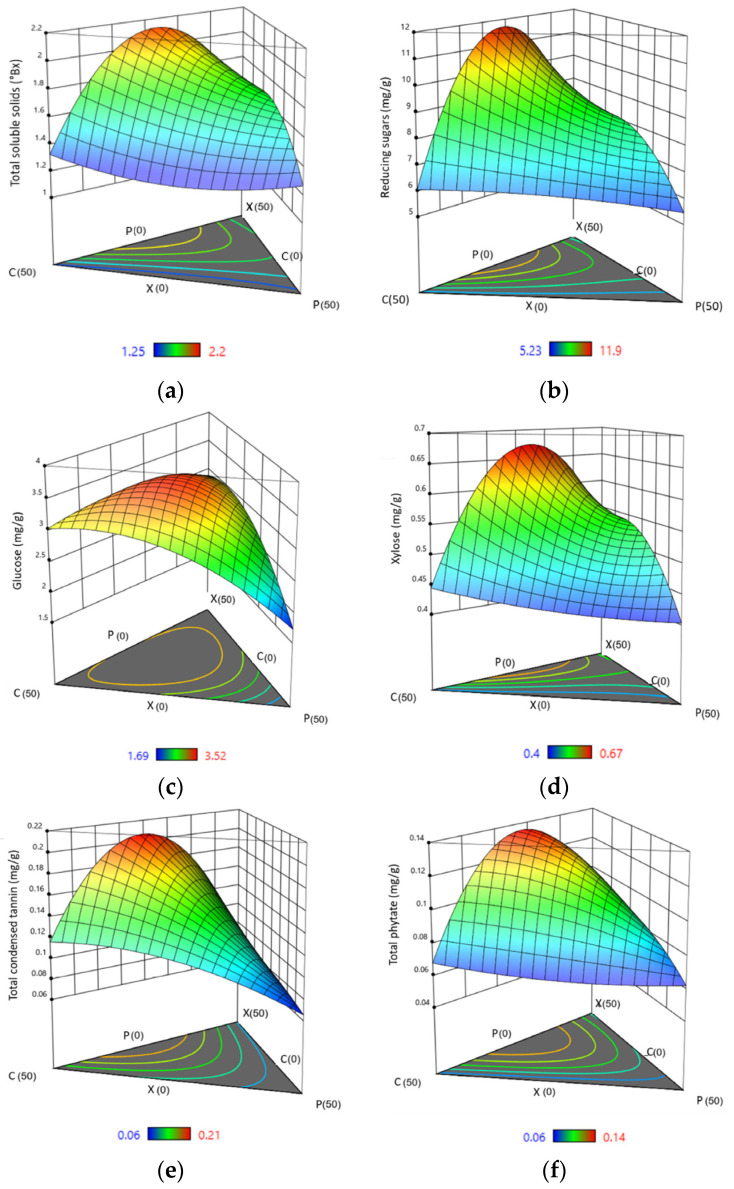
Estimated three-dimensional ternary plots indicating the effect of xylanase, cellulase, and pectinase concentration on (**a**) total soluble solids, (**b**) reducing sugars, (**c**) glucose, (**d**) xylose, (**e**) total condensed tannins, and (**f**) total phytate. X: xylanase, C: cellulase, and P: pectinase.

To better understand the influence of different combinations of enzyme proportions on the yield of hydrolysed sugars and antinutrients, ternary plots were developed (Figure 2) showing the significance of each component of the mixture on the studied response, with contour lines representing the intensity of the effect. It should be noted that the summation of the lengths of perpendiculars from any point in the contour to the three boundaries corresponds to the total sum of the mixture components. Moreover, the outline of the isoline depicts the intensity of different interactions, with an oval representing significant interactions and a round shape denoting insignificance [39]. An elliptical isoline with both major and minor axes represents that the response values are inconsistent with respect to different treatment combinations indicating the presence of interactions. In contrast, a circular isoline with constant axis lengths denotes no interactions.

#### 3.2.1. Total Soluble Solids (TSS)

TSS is an indirect indicator of sugar content in a particular solution, although it also accounts for dissolved organic acids, vitamins, phenols, pectin, etc. [40]. The TSS percentage in the pericarp extract after enzyme treatment indicates the concentration of released sugars upon hydrolysis of the NSPs, which constitutes the major portion of the sorghum bran [41]. The yield of TSS in the pericarp extract after 6 h of incubation ranged from 1.25 to 2.20 °Bx across the experimental enzyme combinations studied (Table 1). The highest yield of 2.20 °Bx was attained when an equal concentration of xylanase and cellulase was used in the cocktail with no addition of pectinase (Table 1). The Scheffe model with quadratic mixture order was found to exhibit significant differences (*p* < 0.05) in TSS, with the interactive effect of xylanase and cellulase significantly affecting the response, showing their importance in the cocktail mix (Table 3). A lower model *p*-value (<0.05) and higher determination coefficient further indicate that the model fits well and could predict the change in TSS of the obtained pericarp extract. The regression model for the experiment showing the highest R-square value (Table 3) and providing the best fit is given in Equation (4):

TSS (°Bx) = 1.47 × X + 1.32 × C + 1.26 × P + 2.93 × X × C + 1.21 × X × P − 0.48 × C × P(4)

where X, C, and P are the individual components of the enzyme mixture representing xylanase, cellulase, and pectinase, respectively.

As seen from the closed-spaced contour lines in the ternary plot in Figure 2a, the proportion of xylanase and cellulase shows a dominant effect. The contribution of pectinase in the release of soluble solids was less than the other enzymes. Figure 2a also shows that the yield substantially decreased on moving vertically down the plot towards equal xylanase and cellulase proportions. This implies that a higher level of TSS can be obtained when the concentration of xylanase and cellulase are almost equal while a lower concentration of pectinase is required. The results also showed that a synergistic interaction resulted in an additive increase in soluble solids when compared to treating with individual enzymes. Hu et al. [42], in their work to determine the possible synergism of xylanase and cellulase for hydrolysing corn stover, sweet sorghum, and lodgepole pine, explained that an increase in fibre porosity and disintegration upon the addition of xylanase in combination with cellulase resulted in shorter fibres and fibre swelling, increasing the cellulose accessibility.

#### 3.2.2. Reducing Sugars (RS)

The concentration of RS in the pericarp extract ranged from 5.23 to 11.96 mg/g, as shown in Table 1. Results from the post hoc test show significant differences among the obtained yields from the different enzyme treatments. Xylanase and cellulase were the major influencing factors, as seen from the higher F-values in Table 3. When xylanase and cellulase were the only mixture components in the cocktail, the RS production was double than it was when pectinase enzyme was included in isolation. Arabinoxylans, β-glucans, and cellulose are the major dietary fibre constituents in sorghum cell walls [43]. Arabinoxylans consist of 1,4-β-d-xylopyranose backbone chains, which are substituted mainly with α-l-arabinofuranose units at O-3 and/or O-3 of certain xylopyranose units. In addition, significant amounts of uronic acid residues, mostly d-glucuronic acid, are attached. The hydrolysis of cellulose, hemicellulose, and pectin-rich bran layers via enzyme action causes the release of these monomeric sugars into the extract, increasing the total yield of reducing sugars. The ANOVA was fitted to the special cubic model, with the interaction of xylanase and cellulase being significant at *p* < 0.01. The design responses were well-fitted into a second-order polynomial equation (Equation (5)), where X, C, and P represent xylanase, cellulase, and pectinase concentrations, respectively.

RS (mg/g) = 6.93 × X + 6.05 × C + 5.45 × P + 20.79 × X × C + 7.36 × X × P + 0.78 × C × P − 18.66 × X × C × P(5)

The isoline outlines from the developed ternary plots (Figure 2b) indicate that a higher proportion of xylanase and cellulase in the cocktail allowed a significantly greater release of RS, whereas pectinase alone yielded minimum results. However, the synergies among xylanase, cellulase, and pectinase promote efficient and better degradation of fibrous pericarp and release of reducing sugars. Lin et al. [44] stipulated that a single enzyme restricted hydrolytic action, whereas a mixed-enzyme complex demonstrated a synergetic impact in lignocellulose hydrolysis, where a hydrolytic activity ratio of the enzyme cocktail was found to be superior to that of the individual enzymes. Additionally, xylanases help remove the hemicellulose barriers, exposing more cellulose chains and increasing the bioconversion of cellulose and hemicellulose. In the same way, Dutta et al. [45] suggested that on pre-treating rice husk and straw samples with xylanase prior to cellulase, the percentage RS increased, as treatment with xylanase might have made the xylan layer more accessible to cellulase.

#### 3.2.3. Glucose

With the observed increase in the yield of RS, an increase in glucose yield is also expected upon enzymatic hydrolysis using the three-enzyme mix. The highest glucose yield under the specified treatment conditions was obtained when cellulase was the major component in the enzyme mixture, with equal proportions of xylanase and pectinase (Table 1). With an overall statistically significant (*p* < 0.05) difference in the means, as displayed in the ANOVA results (Table 3), a post hoc analysis showed significant differences among the different treatment combinations. On comparing various predicted models for glucose yield for the experimental conditions studied, the Scheffe special cubic model was the best-fit, with the highest F-value. Singh et al. [46] optimised enzyme ratios in a cellulase cocktail for saccharification of base pre-treated Sorghum durra stalk and found that the special cubic model was the best-suited model for glucose yield. By looking at the F-value among the variables of mixture components, it is seen that the linear mixture of individual parameters showed the highest F-value, followed by the interactive effect of xylanase and cellulase. The linear term represents the relative effects of all the pure components in the mixture, and the significance (*p* < 0.05) of this term, in contrast to other model terms, indicates that a binary and tertiary mixture yields less glucose in the sample extract relative to that obtained using each enzyme separately. The cellulose content can be hydrolysed into cellobiose and glucose by cellulases, including endoglucanases, cellobiohydrolases, and β-glucosidases, which work together to release glucose [47]. In addition, multi-enzyme mixtures based on xylanase can achieve more refined hydrolysis of pericarp, as cellulose microfibrils are often concealed with other polysaccharides, such as hemicellulose or xyloglucans, and xylanase supplementation; therefore, this improves the performance of cellulase [44]. The variation in glucose yield with a change in enzyme compositions in the cocktail is presented by the following equation generated from the best-fit model:

Glucose (mg/g) = 2.91 × X + 3.02 × C + 1.73 × P + 2.08 × X × C + 0.86 × X × P + 1.81 × C × P + 9.03 × X × C × P (6)

where X, C, and P are the individual components of the enzyme mixture representing xylanase, cellulase, and pectinase, respectively.

From Figure 2c, it is seen that the proportion of cellulase in the cocktail is the major contributing factor to glucose release, followed by xylanase and a slight contribution from pectinase. Cellulase acts on cellulose molecules by hydrolysing the b-1,4 glycosidic linkages and breaking down cellulose, one of the major polysaccharides of sorghum pericarp. It predominantly produces cellobiose, which ultimately yields glucose units. Several other studies have shown that glucose release is improved by xylose removal, increasing cellulose accessibility [48,49].

#### 3.2.4. Xylose

The endosperm and bran layers of sorghum have hemicellulose polysaccharides which are β-1,4-linked pyranosyl units that form hydrogen bonding with cellulose, imparting hydrophobic properties to the cell wall matrix [50]. The main hemicellulose is xylan, a branched polysaccharide with xylose residues connected by β-1,4 glycosidic bonds [48]. Xylose is one of the major sugars found in the NSPs. With tissue softening due to the tempering, induction, and mobilisation of cell wall degrading enzymes, a considerable yield of xylose was found in the sample extract. The maximum yield of xylose in the extract was achieved with equal levels of xylanase and cellulase in the cocktail, and the minimum yield was obtained with pectinase alone (*p* < 0.05) (Table 1). Table 3 shows the interaction effect of xylanase and cellulase following ANOVA (*p* < 0.05). The best fit for the response variable was the special cubic model for the experimental conditions investigated and is described by the following equation:

Xylose (mg/g) = 0.53 × X + 0.44 × C + 0.40 × P + 0.78 × X × C + 0.27 × X × P − 0.04 × C × P − 1.05 × X × C × P(7)

where X, C, and P are the individual components of the enzyme mixture representing xylanase, cellulase, and pectinase, respectively.

The ternary 3D plots for xylose yield developed from the cubic model show regions that outline the highest yield of 0.67 mg/g, obtained by mixtures with a higher proportion of xylanase and cellulase, confirming strong positive synergism between xylanase and cellulase in the partial hydrolysis of sorghum grain pericarp. Xylanases degrade the linear polysaccharide β 1,4-xylane into xylose, thus breaking down hemicellulose, which is a major constituent of the plant cell wall [51].

#### 3.2.5. Galacturonic Acid (GalA)

Although pectin is absent in the sorghum pericarp or is present in significantly lower amounts compared to arabinoxylans and cellulose, the NSPs are composed of pectin-like substances, including uronic acids made up of D-galacturonic acid residues. Galacturonic acid is one of the main monosaccharide components found in sorghum arabinoxylan, reportedly associated with the xylose backbone [52]. Therefore, the presence of GalA in the extract suggests degradation of grain pericarp. However, the model obtained was not significant (Table 3); therefore, these results cannot be confirmed.

#### 3.2.6. Anti-Nutrients

Sorghum is also rich in phenolic compounds such as free, soluble conjugates, and insoluble bound forms. The bran contains a higher percentage of bound phenolics in the components, namely, aleurone layer, testa, and pericarp, while free phenols are found in the vacuoles [53]. About 95% of the grain phenolics are covalently bonded with the cell wall polysaccharides and are signified as dietary fibre phenolic components.

Tannins are highly reactive, unstable phenol derivatives considered antinutritional factors and are found in a higher concentration in some varieties of sorghum than in other cereals [54]. Tannins can be physically, chemically, and biologically degraded to improve the functionality and digestibility of the grain, as shown by the observed concentration of total condensed tannins (TCT) in the enzymatic extract upon soaking the sorghum grains in the enzyme cocktail mixtures. Various factors might have contributed to the release of condensed tannins from the grain pericarp. The use of cell wall degrading enzymes for assisting cereal decortication leads to the development of water-soluble fibre units with the release of phenolic substances [53]. These are also reported to be significantly reduced upon soaking due to the compound’s water-soluble nature, which is mainly concentrated in the grain seed coat. The results presented here showed a significant difference in the yield of TCT obtained from the different experimental enzyme combinations. The Scheffe special cubic model was found to be significant, with the interaction of xylanase and cellulase releasing the most tannins (Table 3). Multiple regression analysis over the entire experimental data created the following 2nd order equation from the best-fit model:

TCT (mg/g) = 0.08 × X + 0.12 × C + 0.06 × P + 0.42 × X × C + 0.02 × X × P + 0.06 × C × P + 0.09 × X × C × P(8)

where X, C, and P are the individual components of the enzyme mixture representing xylanase, cellulase, and pectinase, respectively.

Figure 2e shows the contour ternary plot for TCT yield in the extract obtained by a special cubic model. It is seen that a combination of enzymes gave higher yields compared to individual enzymes, which adds to the importance of a synergistic mixture for grain pericarp hydrolysis.

Phytic acids are stored in the electron-dense spherical particles named globoids, localised mainly in the aleurone layer. Even a slight tear of the aleurone cells could cause the release of phytate into the surrounding medium [55]. Similar to tannins, xylanase–cellulase resulted in the maximum release of phytates, followed by the combination of all three enzymes with a greater proportion of xylanase (Figure 2f). Xylanases are known to hydrolyse arabinoxylans, which constitute the major components of the cell wall in the aleurone layer and are a main storage site of phytates. Xylanases can cause an increase in permeability of the aleurone layer, preferably initiating phytate degradation [56]. Similarly, cellulolytic enzymes are reported to secrete certain hydrolases that can act upon phytate complexes, causing their release into the extract [57]. A regression equation was obtained from the experimental data that predicts the optimal phytate yield with selected parameters:

Phytate (mg/g) = 0.08 × X + 0.07 × C + 0.06 × P + 0.24 × X × C + 0.05 × X × P − 0.02 × C × P + 0.35 × X × C × P (9)

where X, C, and P are the individual components of the enzyme mixture representing xylanase, cellulase, and pectinase, respectively.

Condensed tannins and phytates act as anti-nutrients, and the release of these substances from the grain is thus favourable, increasing the nutrient and mineral bioavailability.

### 3.3. Optimal Enzyme Mixture Composition

Numerical optimisation was conducted to determine the optimum proportion of each enzyme in the cocktail which resulted in the maximum release of pericarp constituents within the decided incubation time. Derringer’s prediction tool was used for this purpose, where the responses were transformed to individual desirability functions and the program set to maximise this function [58]. In addition to the design points, different random points were also checked during the numerical optimisation to obtain the most desirable solution. For the present design, 101 starting points were chosen, and the goal-seeking began at a random point, proceeding up the steepest slope to a maximum. From the multiple solutions obtained, the factor settings that gave the highest desirability score were chosen as the best solution that met the specified criteria, as outlined in Table 4.

Accordingly, the software predicted a cocktail mixture with 33.3 mg/100 g dry matter xylanase and 8.3 mg/100 g dry matter each of cellulase and pectinase could yield the targeted responses with a desirability score of 0.78 when the overall cocktail concentration was kept at 50 mg/100 g dry matter. In these concentrations, the enzyme activities calculated in 20 mL buffer were xylanase: 0.375 U/mL, cellulase: 0.375 U/mL, and pectinase: 0.3 U/mL. Upon validation of the predicted values, it was found that the experimental results were consistent with the predicted ones, with low error percentages indicating the high analytical ability of the mixture design and accuracy of the model. Thus, an enzyme cocktail with 66.7% xylanase and 16.7% each of cellulase and pectinase is recommended for tempering sorghum grains prior to decortication.

### 3.4. Time Course Release of Pericarp Constituents

**Figure 3:**
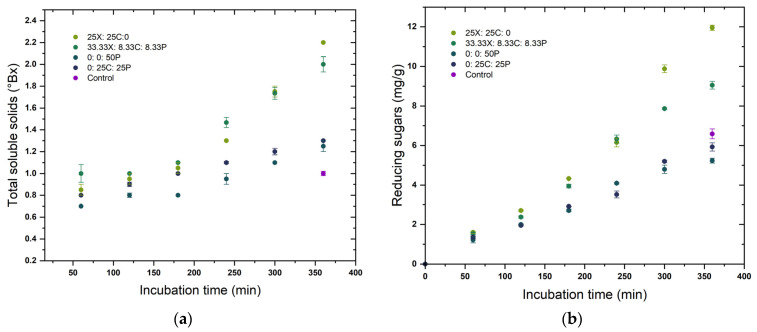

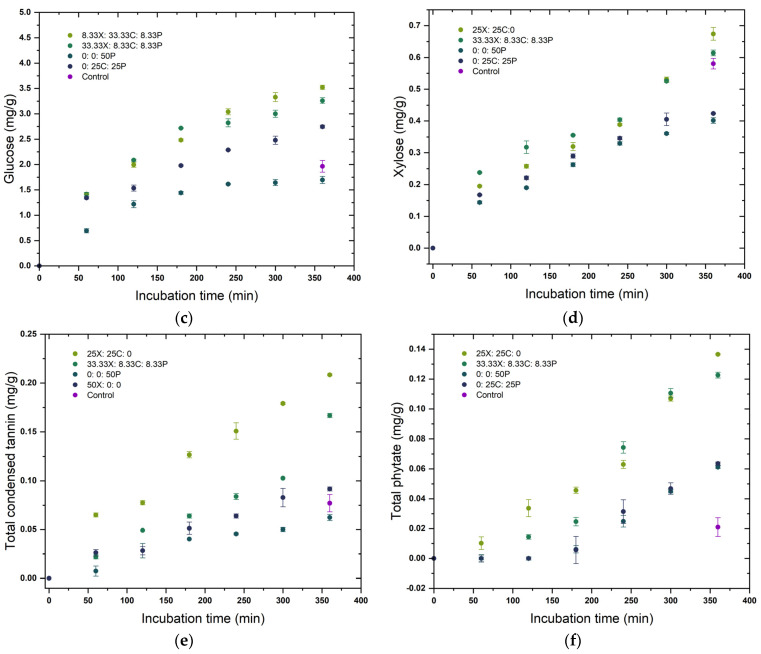
Time course release of pericarp components (**a**) total soluble solids, (**b**) reducing sugars, (**c**) glucose, (**d**) xylose, (**e**) total condensed tannins, and (**f**) total phytate upon treatment with different enzyme cocktail formulations. X: xylanase; C: cellulase, and P: pectinase.

The predicted optimal enzyme concentrations in the mixture that target maximum yield for each pericarp constituent were also assessed in a time-course hydrolysis experiment for 6 h, as shown in Figure 3. Two enzyme combinations that resulted in the maximum yield and two that led to minimum yields of respective pericarp components are depicted, along with the release that occurred upon water soaking the grains for the same treatment time (control). The depicted concentration represents the differential concentration of each component, i.e., concentration at specific time t minus the except for phytates. It can be seen that the release of specific pericarp components was initiated from the beginning of incubation, whereas for phytates the release rate was comparatively low at the beginning. A possible explanation for this could be that phytates are more concentrated in the inner grain layers [16] and its hydrolysis was initiated upon release of other pericarp components from the outer layers. The rate at which specific components were released into the extract showed a gradual decrease towards the end of the incubation period in most of the enzyme combinations, leading to minimum yields of pericarp constituents. However, in the enzyme combinations leading to maximum yields, some pericarp constituents were still increasing at a linear rate at the end of of the experiment. It was presumed that the initial rate of enzymatic hydrolysis depends on the enzyme accessible surface area, while at later stages, the slowdown of hydrolysis was due to the difficulty of hydrolysing the highly crystalline portions of the cellulose [59]. The plots confirm the conclusions from the ANOVA (Table 3) and ternary plots (Figure 2), that a synergistic mixture of three enzymes resulted in a better release of pericarp components than the enzymes applied individually. The model developed for galacturonic acid was not significant (*p* > 0.05) and is therefore not included in Figure 3.

### 3.5. Morphological Characteristics

**Figure 4:**
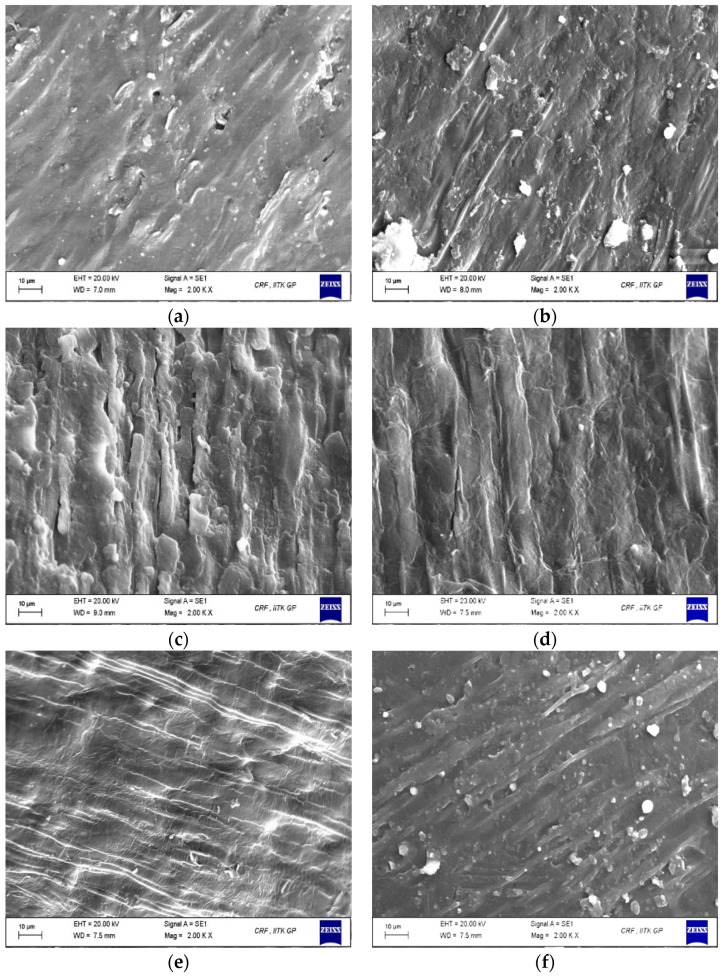
Scanning electron microscopic images of sorghum grains (**a**) untreated, (**b**) water-soaked, (**c**) 33.33X:8.33C:8.333P, (**d**) 25X:25C:0, (**e**) 0:25C:25P, and (**f**) 0:0:50P, where X: xylanase; C: cellulase, and P: pectinase.

From the external to the internal face, the structure of sorghum grain can be partitioned into three major parts: the outer pericarp, the endosperm, and the germ. An efficient decortication process is defined by completely removing the pericarp and much of the germ, which provides a high endosperm recovery with minimum breakage [60]. The surface morphology of sorghum grains, both untreated and treated, at a magnification of 2000× are presented in Figure 4. Figure 4a shows the lateral side of the outer grain surface of the untreated whole sorghum grain with an intact pericarp and a more compact lenticular structure, where the smooth texture in the image can be seen. Upon water soaking (Figure 4b), a certain lack of compactness and creation of intercellular spaces in the pericarp can be seen, indicating the hydration process and microchannel formation resulting from the leaching of pericarp components into the water.

On introducing cell wall degrading enzymes into the tempering buffer, a rougher and more loosened pericarp was observed on the grain surface, which can be seen as a macro-scale indication of enzyme action (Figure 4c–f). The degradation of sorghum pericarp due to the hydrolytic action of the three-enzyme mixture on the NSPs has resulted in a disrupted grain surface with thinned fibrous layers and a torn membrane. The observed changes can be attributed to the activities of xylanase, cellulase, and pectinase enzymes which cause the disintegration of arabinoxylans, cellulose, β-glucan, pectin, and xyloglucans as a result of the breaking of various backbone linkages, leading to changes in pericarp and aleurone layers [61]. Sorghum grains tempered with an enzyme cocktail comprising a higher proportion of xylanase, and equal proportions of cellulase and pectinase showed a more eroded surface than other treatment combinations. The results show the potential of cell wall degrading enzymes to loosen the grain pericarp for facilitating an easier further mechanical decortication. Similar results were observed upon enzymatic decortication of rice with cellulase and xylanase enzymes, where an easy absorption of water was predicted through the bran layers, which could lead to rapid gelatinisation of rice while cooking [20].

### 3.6. X-ray Diffraction Analysis

**Figure 5:**
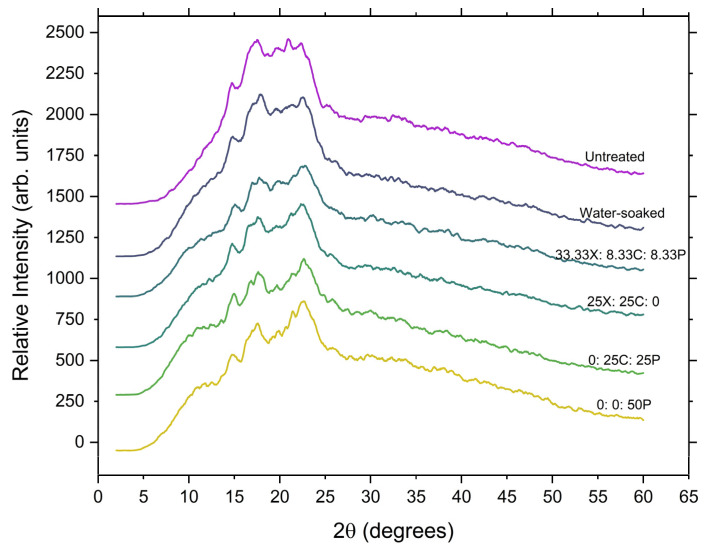
X-ray diffraction patterns of untreated, control (water-soaked), and enzyme-treated grains. X: xylanase; C: cellulase, and P: pectinase.

X-ray diffraction patterns of the whole grain, water-soaked grain, and enzyme-treated samples recorded at a scanning speed of 2°/min are presented in Figure 5. As determined from the diffraction theory, the peak intensity signifies the degree of ordered semi-crystalline structures in the sample. The sharpness of the peak intensifies with the ordered crystalline structure, and peaks become diffused due to the amorphous portions present in the sample. The whole grain diffractograms show the typical A-type diffraction pattern with peaks at 14.8°, 17.4°, and an unresolved triplet around 19.5°–22.2°.

The A-type pattern, characteristic of the whole-grain, was conserved during both water soaking and enzyme tempering (Figure 5), which showed that the cell wall degrading enzymes do not interact with the crystalline structure. When grains are soaked in water, water penetrates the grain, causing progressive swelling, cracking, and destruction of the starch granules [62]. In turn, a partial disruption of the crystalline microstructure of starch occurs, and at this point, the evolution of a new peak at 17.8° can be seen in the control (water-soaked) samples. This is in agreement with Altayan et al., who proposed that the observed growth in this peak is ascribed to B-type crystalline behaviour and can be attributed to the rapid recrystallisation of amylose during long-term moisture exposure [63]. In enzyme-tempered samples, a similar evolution of a peak was noticed, but with less intensity. A major alteration also emerged at the unresolved triplet near 19.5°–22.2°, and the growth in the peak at 22.8° was observed, which also suggests a small extent of preferential hydrolysis of B-type crystalline material [64]. The decrease in relative crystallinity in the samples subjected to enzymatic tempering can be explained as the result of fragmentation of the outer pericarp layers due to enzymatic action. Regardless of the substantial hydrolysis, the X-ray patterns of the enzyme-treated samples remained highly crystalline. This confirms that the enzyme tempering process is highly selective and does not alter the crystalline structure of the starch and that the enzymatic action takes place in the amorphous component of the starch [63]. The A-type arrangement observed for all the study samples is similar to that previously reported in other cereals [63,65,66].

## 4. Conclusions

The results show that each assay of different enzyme cocktail proportions resulted in a different yield of the cell wall constituents in the buffer, indicating that each enzyme acts on specific components of the grain pericarp. The optimisation results suggest that a cocktail comprising a major proportion of xylanase, followed by equal proportions of cellulase and pectinase, could yield a maximum release of pericarp components when the activities were 0.375, 0.375, and 0.3 U/mL for xylanase, cellulase, and pectinase, respectively, as used in this study. A synergistic mixture was found to be more effective in degrading the pericarp than an individual enzyme, which confirms the potential for using enzyme cocktails in hydrolysing cell wall polysaccharides. Since a synergism of enzymes are proven to exert more effect on the substrate than individual enzymes, the proportion of enzymes in the cocktail is a decisive factor that can vary with the composition of the target grains. The study has a substantial impact as far as the decortication or polishing and milling of sorghum is concerned. However, a detailed investigation of the enzyme operating conditions, including concentration, buffer pH, incubation temperature, and incubation time, is required before specific recommendations of the enzyme cocktail to use for sorghum decortication can be given.

## Figures and Tables

**Table 1:** Statistical mixture experimental design, showing the combinations of individual enzymes, xylanase (X), cellulase (C), and pectinase (P), in the cocktail and the corresponding concentration of the released components from the pericarp as analysed following enzymatic pre-treatment.

Runs	Enzyme Cocktail Proportions (mg/100 g dm)			TSS (°Bx)	RS (mg/g)	Glucose (mg/g)	Xylose (mg/g)	GalA (mg/g)	TCT (mg/g)	Phytate (mg/g)
	X	C	P							
1	0	0	50	1.25 ± 0.05 ^a^	5.23 ± 0.12 ^a^	1.70 ± 0.03 ^a^	0.40 ± 0.001 ^a^	0.21 ± 0.006	0.06 ± 0.003 ^a^	0.06 ± 0.001 ^a^
2	0	25	25	1.30 ± 0.00 ^a,b^	5.92 ± 0.01 ^b^	2.75 ± 0.02 ^b^	0.42 ± 0.001 ^b^	0.25 ± 0.002	0.10 ± 0.003 ^c,d^	0.06 ± 0.001 ^a^
3	0	50	0	1.35 ± 0.05 ^a,b,c^	6.24 ± 0.12 ^c^	2.97 ± 0.03 ^c^	0.46 ± 0.006 ^c^	0.18 ± 0.001	0.11 ± 0.00 ^d^	0.07 ± 0.007 ^b^
4	8.33	8.33	33.3	1.45 ± 0.05 ^c,d^	7.52 ± 0.04 ^e^	2.92 ± 0.02 ^c^	0.46 ± 0.005 ^c,d^	0.22 ± 0.008	0.11 ± 0.003 ^d^	0.08 ± 0.001 ^c,d^
5	8.33	33.3	8.33	1.50 ± 0.04 ^d,e^	7.72 ± 0.05 ^e,f^	3.52 ± 0.04 ^f^	0.48 ± 0.007 ^d^	0.17 ± 0.004	0.17 ± 0.005 ^g^	0.09 ± 0.002 ^d^
6	16.67	16.67	16.67	1.60 ± 0.02 ^e,f^	8.83 ± 0.10 ^g^	3.24 ± 0.03 ^d,e^	0.53 ± 0.002 ^e^	0.22 ± 0.002	0.15 ± 0.01 ^f^	0.12 ± 0.001 ^e^
7	25	0	25	1.70 ± 0.00 ^f^	7.89 ± 0.17 ^f^	2.83 ± 0.01 ^b^	0.52 ± 0.01 ^e^	0.19 ± 0.001	0.08 ± 0.002 ^b^	0.08 ± 0.002 ^c,d^
8	25	25	0	2.20 ± 0.01 ^h^	11.96 ± 0.01 ^h^	3.16 ± 0.05 ^d^	0.67 ± 0.01 ^g^	0.15 ± 0.002	0.21 ± 0.001 ^h^	0.14 ± 0.004 ^f^
9	33.3	8.33	8.33	2.00 ± 0.09 ^g^	9.05 ± 0.05 ^g^	3.26 ± 0.02 ^e^	0.61 ± 0.002 ^f^	0.20 ± 0.006	0.13 ± 0.002 ^e^	0.12 ± 0.001 ^e^
10	50	0	0	1.40 ± 0.03 ^b,c,d^	7.01 ± 0.10 ^d^	2.93 ± 0.01 ^c^	0.51 ± 0.003 ^e^	0.16 ± 0.005	0.09 ± 0.007 ^b,c^	0.08 ± 0.002 ^b,c^

Values with different letter superscripts in the same column were significantly different at *p* < 0.05 as per Tukey’s post hoc analysis.

**Table 2:** Proximate composition, mineral content, total phenols, and antinutrient content of sorghum flour obtained from whole unpolished and conventionally decorticated grains to show the loss of macro- and micro-nutrients.

Parameter	Whole Grain Flour	Decorticated Grain Flour
Moisture content (%)	10.10 ± 0.07	8.59 ± 0.06
Protein (g/100 g)	11.59 ± 0.30	5.50 ± 0.01
Fat (g/100 g)	3.09 ± 0.21	2.46 ± 0.07
Ash (g/100 g)	2.39 ± 0.18	1.56 ± 0.14
Crude fibre (g/100 g)	7.17 ± 0.45	5.45 ± 0.24
Carbohydrates (g/100 g)	65.65 ± 0.31	76.44 ± 0.48
Dietary fibre (g/100 g)	35.20 ± 0.03	25.44 ± 0.08
Iron (mg/100 g)	5.65 ± 0.42	3.33 ± 0.23
Zinc (mg/100 g)	4.02 ± 0.14	1.93 ± 0.06
Total phenols (mg GAE/100 g)	39.32 ± 0.03	34.29 ± 0.01
Total condensed tannin (mg CE/g)	5.43 ± 0.13	1.44 ± 0.03
Total phytates (mg/g)	17.93 ± 0.02	15.79 ± 0.03

Results are expressed as mean values ± standard deviations on an as-is basis.

**Table 3:** Results of two-way analysis of variance (ANOVA) showing Fisher’s F-statistic and model significance of different proportions of individual enzymes in the mixture on the yield of pericarp constituents from sorghum grain following enzymatic pretreatment.

Model Terms	TSS (°Bx)	RS (mg/g)	Glucose (mg/g)	Xylose (mg/g)	GalA (mg/g)	TCT (mg/g)	Phytate (mg/g)
Model	6.59	15.48	9.44	9.35	3.15	16.95	11.76
Linear mixture	5.45	14.98 *	17.22 *	14.96 *	5.11	21.58 *	11.44 *
XC	18.45 *	52.79 **	5.00	22.69 *	0.358	46.23 **	30.23 *
XP	3.16	6.63	0.868	2.81	0.66	0.077	1.33
CP	0.490	0.076	3.80	0.072	6.33	1.06	0.157
XCP	-	0.979	2.17	0.998	-	0.056	1.49
*p*_model_	0.0458 *	0.0233 *	0.0465 *	0.0470 *	0.124 ^ns^	0.0205 *	0.0343 *
*R* ^2^	0.892	0.969	0.949	0.949	0.474	0.971	0.959
*Adj R* ^2^	0.756	0.906	0.849	0.84	0.324	0.914	0.878

* 5% level of significance, ** 1% level of significance, ns: not significant; *Adj*: adjusted, X: xylanase, C: cellulase, P: pectinase, TSS: total soluble solids, RS: reducing sugars, GalA: galactouronic acid, TCT: total condensed tannins.

**Table 4:** Results of numerical optimisation to determine the optimal proportion of each enzyme in the cocktail, showing constraints, importance, predicted, and experimental values of the proportion of xylanase, cellulase, and pectinase in the enzyme cocktail, and the corresponding responses on the yield of pericarp constituents from sorghum grain following enzymatic pretreatment following Derringer’s prediction tool.

Parameter	Goal	Importance	Predicted Value (D = 0.78)	Experimental Value	Relative Error (%)
Xylanase (mg/100 g dm)	In range	-	33.3	33.3	-
Cellulase (mg/100 g dm)	In range	-	8.3	8.3	-
Pectinase (mg/100 g dm)	In range	-	8.3	8.3	-
TSS (°Bx)	Max	3	1.86	1.96 ± 0.04	5.10
Reducing sugars (mg/g)	Max	3	9.34	9.19 ± 0.11	1.63
Glucose (mg/g)	Max	3	3.27	3.22 ± 0.03	1.55
Xylose (mg/g)	Max	3	0.58	0.60 ± 0.03	3.33
Galacturonic acid (mg/g)	-	-	0.18	0.19 ± 0.01	5.26
Total condensed tannins (mg/g)	Max	3	0.14	0.14 ± 0.01	0.71
Total phytate (mg/g)	Max	3	0.12	0.11 ± 0.01	6.20

## Data Availability

The data is included in the article.

## Appendix A

**Table A1:** Enzyme activities of xylanase, cellulase, and pectinase in different combinations of enzyme cocktail with an overall cocktail concentration of 50 mg/100 g dry matter.

Runs	Cocktail Combinations with Overall Enzyme Concentration = 50 mg/100 g dm	Enzyme Activities (U/mL)		
		Xylanase	Cellulase	Pectinase
1	0:0:50P	0	0	1.8
2	0:25C:25P	0	1.125	0.9
3	0:50C:0	0	2.25	0
4	8.33X:8.33C:33.33P	0.094	0.375	1.2
5	8.33X:33.33C:8.33P	0.094	1.5	0.3
6	16.67X:16.67C:16.67P	0.188	0.75	1.2
7	25X:0:25P	0.281	0	0.9
8	25X:25C:0	0.281	1.125	0
9	33.33X:8.33C:8.33P	0.375	0.375	0.3
10	50X:0:0	0.562	0	0
